# Tryptophan Metabolism in Pediatric Atopic Dermatitis: Shared Mechanisms, Age-Specific Biomarkers, and Emerging Therapies

**DOI:** 10.3390/biomedicines14102140

**Published:** 2026-09-22

**Authors:** Marta Dyszkiewicz, Beata Cudowska, Wioleta Justyna Omeljaniuk, Sylwia Marcinkiewicz-Rybołowicz, Dariusz Marek Lebensztejn

**Affiliations:** 1 Department of Pediatrics, Gastroenterology, Hepatology, Nutrition, Allergology and Pulmonology, Faculty of Medicine, Medical University of Bialystok, Jana Kilinskiego, 115-089 Bialystok, Poland; beata.cudowska@umb.edu.pl (B.C.); sylwia.marcinkiewicz-rybolowicz@umb.edu.pl (S.M.-R.); dariusz.lebensztejn@umb.edu.pl (D.M.L.); 2 Department of Analysis and Bioanalysis of Medicines, Faculty of Pharmacy with the Division of Laboratory Medicine, Medical University of Bialystok, Mickiewicza Street 2D, 15-222 Bialystok, Poland; wioleta.omeljaniuk@umb.edu.pl

**Keywords:** tryptophan, kynurenine pathway, aryl hydrocarbon receptor, pediatric atopic dermatitis, biomarkers, endotypes and phenotypes, gut–skin microbiome, biologic therapies, precision medicine, allergic diseases

## Abstract

Atopic dermatitis affects up to one in five children; however, most mechanistic evidence linking tryptophan metabolism to disease pathogenesis derives from adult cohorts and preclinical models. Whether these mechanisms operate similarly across pediatric developmental stages remains poorly characterized. Tryptophan metabolism, through the kynurenine, serotonin–melatonin, and microbiota-derived indole pathways, regulates immune homeostasis, epidermal barrier integrity, and gut–skin microbiome crosstalk, with disruptions implicated in pediatric and adult disease alike. This review synthesizes pediatric evidence on tryptophan metabolism in atopic dermatitis, benchmarked against the more extensive adult and preclinical literature, spanning aryl hydrocarbon receptor signaling, filaggrin regulation, gut and skin microbiome-derived metabolites, circulating biomarkers, and emerging therapeutics including tapinarof, melatonin, and dupilumab. Age-stratified evidence indicates that components of this pathway have clinical or translational feasibility in children; however, standardized, prospective studies directly contrasting age groups are still lacking. By mapping pediatric–adult differences, the review identifies mechanisms that may be shared across age groups while acknowledging the limited direct pediatric evidence, providing a hypothesis-generating framework for future age-appropriate, biomarker-informed precision medicine in pediatric atopic dermatitis (PAD).

## 1. Introduction

Atopic dermatitis (AD) is the most common chronic inflammatory skin disease in childhood, affecting up to one in five children in high-income countries and imposing a substantial burden on the quality of life of both patients and caregivers [1,2,3,4,5,6,7,8]. What was once conceptualized as a single disease entity is now understood to be a markedly heterogeneous condition, encompassing multiple clinical phenotypes (early-onset versus late-onset, intrinsic versus extrinsic, flexural versus non-flexural) that overlie distinct molecular endotypes defined by differential activation of Th2, Th17, and Th22 immune axes [9,10]. This heterogeneity has direct clinical consequences, as patients with different endotypes may respond differently to the same targeted therapies [11,12].

The recognition of this heterogeneity has driven a shift toward precision dermatology, which aims to match individual patients to the therapy most likely to benefit them, guided by objective, measurable biomarkers rather than clinical phenotype alone [13,14]. This ambition is particularly urgent in PAD, where the immune and cutaneous phenotype may differ from that of adults [15,16,17,18], yet validated, age-appropriate biomarkers remain markedly scarcer than in the adult literature [11,19].

This review positions tryptophan metabolism, encompassing the kynurenine, serotonin–melatonin, and microbial indole pathways, as an underexplored, mechanistically well-grounded axis with potential relevance for biomarker development in PAD. It traces the evidence from foundational adult and preclinical mechanistic studies to the smaller but emerging body of pediatric-specific data. Reference [20] surveys this mechanistic landscape broadly across the AD literature without a specific pediatric focus. In contrast, this review focuses on the pediatric evidence base, systematically mapping the available data and highlighting the gaps that remain to be addressed in future pediatric biomarker research.

### 1.1. Age-Stratified Definitions and Immunological Comparison Across the Pediatric–Adult Spectrum

An important consideration in interpreting PAD literature is that ‘pediatric’ encompasses biologically distinct developmental stages. Infant, child, and adolescent skin and immune biology differ significantly, and several of the transcriptomic studies this review relies on for its pediatric-specific claims explicitly compare across these subgroups rather than treating them as equivalent; see Table 1.

### 1.2. Pediatric-Specific Barrier Maturation Evidence

The infant age band’s rationale above is directly supported by dedicated pediatric cohorts: filaggrin, the structural protein central to epidermal barrier integrity and a major genetic risk factor for AD across the lifespan [21], shows loss-of-function variants with age- and cohort-specific associations with AD phenotype in a diverse, early-life prospective cohort [22], and the PreventADALL birth cohort similarly links filaggrin mutations to skin-barrier function specifically in early infancy [23], reinforcing that barrier maturation in the 0–2-year window is not a theoretical construct but an empirically documented, age-specific process distinct from adult barrier biology.

Direct cross-age-group evidence. A small but growing set of studies directly compares immunological/transcriptomic profiles across these bands within the same analytic framework, rather than studying a single age band in isolation (Table 2):

### 1.3. Age-Specific and Developmental Factors

Atopic dermatitis is substantially more prevalent in early childhood than at any later life stage, a pattern that follows directly from the biology of the developing skin and immune system rather than from chance. The infant and early-childhood skin barrier is structurally and functionally immature: the stratum corneum is thinner, natural moisturizing factor content is lower, and transepidermal water loss is correspondingly higher than in adult skin [21,22,23]. Baseline filaggrin expression is also lower in early life, independent of FLG mutation status, further reducing barrier resilience [22]. This barrier vulnerability coincides with a Th2-skewed early-life immune profile and a gut and skin microbiome that is still maturing during the first years of life [25], together lowering the threshold for allergen penetration, barrier breakdown, and allergic sensitization. As children age, the barrier matures, the immune profile gradually shifts away from Th2 dominance toward a more Th1/Th17-balanced pattern, and the microbiome stabilizes; this progression parallels the well-documented decline in AD prevalence and the change in clinical phenotype from childhood into adolescence and, ultimately, adulthood.

Future pediatric studies of tryptophan metabolism should account for age-related and developmental factors that may independently influence tryptophan-pathway metabolites and complicate their interpretation as biomarkers of atopic dermatitis (AD). Relevant sources of variation include breastfeeding versus formula feeding, which can shape the infant gut microbiome and microbial indole production; antibiotic exposure, which may disrupt indole-producing microbial communities and has been associated with increased AD risk [25]; and the normal maturation of the gut microbiome during the first two to three years of life, which substantially affects baseline microbial metabolic activity. Dietary tryptophan intake may also vary with age and is rarely standardized in PAD metabolomic studies. Importantly, recent prospective longitudinal evidence indicates that fecal tryptophan metabolism may predict the subsequent development of infant eczema, suggesting that alterations in the tryptophan–microbiome axis may precede rather than simply reflect clinical disease [26].

Potential sources of variation also extend to the prenatal period. Maternal tryptophan-pathway metabolism changes dynamically during pregnancy as part of physiological immune adaptation [27], while higher maternal serum nicotinamide and related tryptophan-pathway metabolites in late pregnancy have been associated with a lower risk of offspring atopic eczema at 12 months [28]. Maternal metabolic status may therefore represent an upstream factor influencing offspring susceptibility before postnatal factors such as feeding, antibiotic exposure, diet, and microbiome maturation become relevant.

### 1.4. Literature Search Methodology

Evidence for this review was identified through searches of PubMed/MEDLINE and Scopus/Web of Science for English-language publications from 2007 to 2026, combining terms for the tryptophan-metabolic axis (tryptophan, kynurenine, aryl hydrocarbon receptor/AHR, serotonin, melatonin, indole) with terms for the disease and population of interest (atopic dermatitis, eczema, pediatric/paediatric, child, infant, adolescent, biomarker, endotype), supplemented by hand-searching the reference lists of retrieved reviews. Records were screened by a single author for relevance to tryptophan-pathway metabolism (kynurenine, serotonin–melatonin, or microbial indole branches) in the context of atopic dermatitis or closely related allergic/immunological biology, and records with no plausible mechanistic or clinical link to this axis were excluded. Evidence was stratified by age (pediatric, <18 years; adult, ≥18 years; or mixed/multiple-age) and by evidence type, with preclinical (animal and in vitro/experimental) studies reviewed separately and denoted throughout as mechanistic support rather than direct clinical confirmation, and one preprint explicitly flagged as not yet peer-reviewed.

## 2. Biomarker Landscape in Pediatric AD

Before turning to tryptophan, it is useful to briefly situate it against the biomarkers already used or studied in PAD. These vary considerably in validation status: TARC/CCL17 is the most clinically established, with a validated commercial assay and reproducible correlation with disease severity; the remainder are research-stage or exploratory markers not yet supported by equivalent clinical validation.

- Clinically validated. TARC/CCL17, a Th2-associated chemokine, is among the most consistently validated severity biomarkers in both infant and PAD cohorts, correlating with disease activity even in infants under six months of age [29,30]; serum chemokine levels, including TARC, have separately been shown to track AD severity and allergic sensitization status specifically in children [31].
- Exploratory/mechanistic. IL-13, a central Th2 effector cytokine driving barrier dysfunction and pruritus; the direct pharmacological target of tralokinumab and lebrikizumab; a pharmacological/mechanistic target rather than a validated diagnostic biomarker in its own right.
- Exploratory. Periostin, a matricellular protein induced by IL-13/IL-4 signaling in keratinocytes, proposed as a serum surrogate of local Th2 activity; not yet validated as a standalone clinical biomarker in PAD.
- Exploratory/research-stage. Circulating microRNAs, emerging cell-free biomarkers profiled specifically in PAD cohorts, including serum miRNA-155 as a candidate disease biomarker [32], paired serum/urinary microRNA panels in children with AD [33], and cross-sectional Th1/Th2/Th17 and innate immune biomarker panels in pediatric alopecia areata with and without concurrent AD, offering a comparator dataset for shared versus AD-specific immune signatures [34].
- Peripheral eosinophilia and total/specific IgE, long-standing but not validated as AD-specific markers, have limited standalone diagnostic value but are still widely used in stratification and reported systematically across infant biomarker panels [35].

Across this validation spectrum, these markers share a common limitation: they are largely downstream readouts of Th2 inflammation and provide little direct information about the barrier–microbiome interface or about metabolic/immunometabolic processes that may precede and sustain that inflammation. This is the gap that a tryptophan-centered framework is positioned to address.

## 3. Why Focus on Tryptophan?

Tryptophan is an essential amino acid: it cannot be synthesized de novo and must be obtained from the diet, after which it is partitioned across three principal catabolic routes: the kynurenine pathway (accounting for the large majority of dietary tryptophan catabolism), the serotonin–melatonin pathway, and microbial conversion to indole derivatives in the gut and on the skin [36,37,38].

Three features make tryptophan metabolism mechanistically distinctive as a candidate axis in PAD.

### 3.1. Immunometabolism

Kynurenine-pathway enzymes, chiefly indoleamine 2,3-dioxygenase 1 (IDO1), are directly inducible by inflammatory cytokines (notably IFN-γ) and, in turn, their metabolite products feed back to shape T-cell differentiation and dendritic cell tolerogenicity, placing tryptophan catabolism at the intersection of metabolism and adaptive immunity rather than downstream of it [39,40,41,42].

### 3.2. Microbiome Dependence

A substantial fraction of tryptophan’s immunologically active metabolites—indole, indole-3-aldehyde, indole-3-lactic acid, and indole-3-propionic acid—are not produced by host enzymes at all, but by commensal bacteria, making tryptophan metabolism one of the most direct biochemical readouts of the gut–skin microbiome axis implicated in AD pathogenesis [43,44,45,46].

### 3.3. Convergence on the Aryl Hydrocarbon Receptor (AhR)

Kynurenine-pathway metabolites (kynurenine, kynurenic acid) and microbial indoles converge on a single, druggable receptor, AhR, a ligand-activated transcription factor that also responds to a broader range of dietary and nutritional ligands beyond tryptophan derivatives [47], that is itself essential for keratinocyte differentiation, filaggrin expression, and skin-barrier integrity, and is now recognized as a central regulator of skin homeostasis [48] and a validated therapeutic target in AD [44,49,50,51,52], with natural compounds beyond tapinarof increasingly explored as alternative AhR modulators for AD management [53]. This convergence gives tryptophan metabolites biological plausibility beyond simple correlation, since they sit on a pathway with a direct, targetable mechanistic link to barrier function; whether this mechanistic link translates into diagnostic, prognostic, or predictive value, however, remains to be established, as none of it has yet been formally validated for these purposes in PAD. These features distinguish tryptophan metabolism from the largely cytokine-centric biomarkers reviewed in Section 2 and motivate its dedicated treatment here.

## 4. Tryptophan-Metabolic Pathways

### 4.1. Kynurenine Pathway

Of the three tryptophan catabolic routes, the kynurenine pathway is the most extensively characterized, with metabolites implicated in a broad range of physiological and immune processes beyond the skin [54,55]. The pathway is initiated by oxidative cleavage of the tryptophan indole ring, catalyzed by two rate-limiting enzymes with distinct regulation: indoleamine 2,3-dioxygenase 1 (IDO1), which is broadly tissue-expressed and strongly inducible by inflammatory signals (particularly IFN-γ and, in atopic skin, by Th2/Th17 cytokines), and tryptophan 2,3-dioxygenase (TDO), which is predominantly hepatic and constitutively active, governing systemic tryptophan homeostasis under baseline conditions [41,56].

Downstream of this initial step, kynurenine is further metabolized to kynurenic acid, 3-hydroxykynurenine, and ultimately to quinolinic acid and NAD^+^ via the de novo pathway, with individual branch-point enzymes exerting distinct, sometimes opposing, immunomodulatory effects: kynurenine and kynurenic acid are AhR ligands with predominantly immunoregulatory/tolerogenic effects [57,58], whereas dysregulated flux through this pathway has been linked to pro-inflammatory states in psoriasis and other inflammatory dermatoses via the enzyme L-kynureninase [59].

It should be noted that the L-kynureninase finding above [59], along with several other AhR/kynurenine-pathway mechanistic studies cited in this review, was generated in psoriasis rather than AD, or in barrier biology models not specific to either condition. AD and psoriasis share a compromised skin barrier and cutaneous inflammation but differ fundamentally in their dominant immune axis (AD: Th2/Th17/Th22-skewed; psoriasis: Th17/Th1-skewed with minimal Th2 contribution), so a mechanism validated in psoriasis cannot be assumed to translate unchanged to AD.

The kynurenine/tryptophan ratio (Kyn/Trp) is the most widely used composite readout of kynurenine-pathway flux in the wider immunometabolism literature and is directly measurable in serum or plasma; it is a surrogate of net pathway flux rather than a direct measure of IDO1 activity specifically, since it is also shaped by TDO activity and by systemic metabolic factors (e.g., nutritional status, hepatic function, inflammation-driven changes in amino acid handling) that are not specific to the AhR-relevant immune pathway; one of the few pediatric allergic-disease studies to report IDO activity alongside neopterin (a marker of IFN-γ-driven monocyte/macrophage activation) found both elevated in children with allergic disease, supporting kynurenine pathway activation as a measurable feature of pediatric atopic immune activation; the study also reported an AD-specific subgroup with significantly higher neopterin, tryptophan, and kynurenine levels and higher IDO1 activity than controls [60]. A second, longitudinal study published in early 2026 corroborates this from the indole side of the pathway: in a prospective cohort of 40 infants, fecal tryptophan and tryptamine were elevated in early-onset eczema by 6 months, while indole-3-lactic acid was reduced ahead of late-onset eczema diagnosis, with several tryptophan/indole ratios reaching AUC > 0.8 for discriminating eczema trajectory [26]. Together, refs. [26,60] are the only two studies identified in the literature search described in Section 1.4 that offer an AD-specific, tryptophan-pathway-targeted pediatric signal (Table 3).

### 4.2. Serotonin Pathway

A smaller fraction of dietary tryptophan is diverted toward serotonin synthesis via tryptophan hydroxylase, with serotonin subsequently acetylated and methylated to melatonin. Both serotonin and melatonin have established, though less extensively characterized, roles in cutaneous immune regulation. Beyond its classical neurotransmitter function, serotonin is now recognized as a broader immunomodulator acting directly on multiple immune cell types [66]: it activates human monocytes and modulates their apoptotic threshold [67], and serotonergic markers (transporter expression, receptor density) are altered in the skin of AD patients [68,69], correlating with pruritic and vascular responses [70]. A 5-HT1a receptor agonist has shown efficacy in adult AD in a clinical study [71], and SSRIs have been proposed, though not established, as an adjunctive approach to itch-driven inflammatory skin disease [72,73]. Clinically, lower serum serotonin has also been associated with depressive symptoms in adult AD patients, suggesting the serotonergic branch of this pathway may additionally intersect with the well-documented psychiatric comorbidity burden of AD [74].

Melatonin has the most direct pediatric evidence within this sub-pathway. In a double-blind, placebo-controlled crossover RCT, 48 children and adolescents (aged 1–18 years) with AD received melatonin or placebo for 4 weeks, followed by a 2-week washout and crossover; 38 participants completed the study. Melatonin reduced SCORAD by 9.1 points compared with placebo [75], providing one of the strongest direct pediatric clinical data points within the tryptophan–AD literature. Mechanistically, increased skin microbiota-derived propionic acid has been proposed as an additional effect of melatonin, based on an experimental AD model rather than pediatric human data. This mechanism therefore remains unconfirmed in children treated with melatonin [76,77,78].

### 4.3. Microbial Indole Pathway

The third route, bacterial conversion of unabsorbed dietary and luminal tryptophan into a family of indole derivatives (indole, indole-3-aldehyde, indole-3-lactic acid, indole-3-propionic acid, indole-3-carbinol, and others), is mechanistically the most consequential for AD and is the pathway on which this review’s precision-medicine argument principally rests.

Skin-barrier effects. A skin-microbiota-derived tryptophan metabolite has been shown to attenuate cutaneous inflammation in an AD model through AhR signaling [43], and commensal skin bacteria more broadly regulate barrier repair via the same receptor [50], with skin microbiota-derived indole-3-propionic acid independently shown to improve barrier function in a murine AD model [79]. A 50-species gnotobiotic model of the human skin microbiome directly demonstrated that commensal-derived tryptophan metabolites fortify the skin barrier [80], while indole-3-lactic acid specifically normalizes barrier-relevant gene expression in IL-4/IL-13-stimulated human skin-equivalent AD models [81]. A related indole derivative, indole-3-carbinol, alleviates allergic skin inflammation via suppression of the periostin/TSLP axis, providing an additional, mechanistically distinct route by which indole metabolites limit Th2-driven barrier disruption [82]. In a murine AD model, indole-3-aldehyde and indole-3-lactic acid rebalanced the Th1/Th2 axis (reducing IL-4, IL-13, and TSLP; increasing IFN-γ) via AhR signaling and alleviated AD-like inflammation, reinforcing this same mechanistic pathway with a distinct pair of indole metabolites [83].

Gut–skin axis. The gut–skin axis is now a well-established conceptual framework in AD pathogenesis [84], and murine studies confirm that experimentally induced AD is accompanied by concordant shifts in intestinal flora and their tryptophan-derived metabolites [85]. Bifidobacterium longum improves experimental AD via tryptophan metabolism acting through the gut–skin axis [45], and *Limosilactobacillus reuteri* attenuates AD through comparable changes in indole derivatives [86]; kynurenine itself, when supplied as a probiotic-derived metabolite, has similarly been shown to ameliorate AD in a murine model, extending this gut–skin translational evidence from the indole branch to the kynurenine branch of tryptophan metabolism [87]. A network meta-analysis of probiotic strains in PAD supports clinical benefit of selected strains, though without mechanistic attribution to tryptophan metabolism specifically [88]. A case–control study directly comparing oral, skin, and gut microbiota composition in children with AD provides pediatric-specific support for this gut–skin axis framework, though without targeted tryptophan-pathway metabolite quantification [89]. Indoles more broadly exert dual, context-dependent effects on human health via intestinal microbiota [46], and indole-3-aldehyde specifically restores epithelial/intestinal barrier integrity through AhR in non-cutaneous models, establishing a mechanistic precedent directly transferable to skin [90,91].

The founding mechanistic paper for this entire sub-field remains Zelante et al.’s demonstration that microbiota-derived tryptophan catabolites engage AhR and balance mucosal reactivity via IL-22 [44]—a mechanism subsequently extended from the gut to the skin by the AhR-keratinocyte biology literature and now under direct pharmacological exploitation by AhR-agonist therapeutics (Figure 1).

## 5. Evidence in Pediatric AD and Research Gaps

Direct, tryptophan-pathway-focused metabolomic evidence in PAD remains limited. Although several pediatric metabolomic studies have characterized broader metabolic alterations, only a small number have directly investigated tryptophan-derived metabolites or kynurenine-pathway activity. This scarcity represents a central evidence gap motivating the present review and is consistent with the broader observation that metabolomic characterization of childhood atopic disease remains comparatively underdeveloped relative to adult metabolomic phenotyping [92]. Table 3 summarizes pediatric studies identified in the literature search that provide direct or potentially relevant evidence regarding tryptophan metabolism, together with adult human comparator studies; preclinical (animal and in vitro) evidence for this pathway is discussed narratively in Section 3 and Section 4 and is not tabulated here. Complementing this metabolomic evidence, a non-invasive diagnostic approach based on fecal microbiota and metabolite profiles has recently been reported specifically in PAD [93], illustrating a stool-based alternative to serum sampling that may be particularly well suited to the microbiome-dependent indole arm of the tryptophan pathway (Section 4.3) in young children.

There is also a design problem underlying nearly all of this: the human data summarized above are overwhelmingly cross-sectional, and the field’s mechanistic case still leans heavily on murine models (Section 3 and Section 4). All but one of the pediatric metabolomic studies in Table 3 are cross-sectional single-time-point measurements; only [26] is longitudinal, and even that study’s small subgroups (*n* = 7–12) and purely observational design leave the state-versus-trait and causal-precedence questions below only partly resolved. Outside AD, tryptophan and gut-microbiota-derived metabolites have proven diagnostically informative in another pediatric condition (migraine), establishing methodological precedent that pediatric tryptophan-pathway biomarker panels are technically feasible and can achieve clinically meaningful discrimination when appropriately designed [94], a precedent this review argues has not yet been systematically applied to PAD. This leaves two problems unresolved simultaneously. First, without repeated measures across disease flares and remission in the same children, the field cannot distinguish state markers (reflecting current disease activity) from trait markers (reflecting stable predisposition), a distinction foundational to any diagnostic or predictive biomarker claim. Second, the direction of effect is unknown: does altered tryptophan-pathway activity contribute causally to barrier dysfunction and inflammation, or does it instead reflect barrier dysfunction that is already present, via increased bacterial/metabolite translocation across a compromised barrier, or via inflammation-driven IDO1 induction, making the metabolic change a consequence rather than a driver. We identified no mechanistic study bridging the gap between murine causal models and human observational correlation with an appropriately designed human interventional or randomization study. This ambiguity does not undermine the biomarker case for tryptophan metabolites (a consequence can still be diagnostically or prognostically useful), but it does materially limit any claim that tryptophan-pathway modulation would be therapeutically effective as a primary intervention, as opposed to modulation via upstream barrier- or microbiome-directed approaches or via biologics that normalize inflammation first (Section 7).

Remaining research gaps. Pulling this together with the causal-direction problem discussed above, eight gaps stand out as the field’s principal unresolved priorities:

- No integrated panel. The full tryptophan panel, kynurenine, serotonin/melatonin, and microbial indoles together, has not been measured in the same PAD cohort by any study identified here. Existing data are fragmented across single-pathway studies (Table 3), so which arm contributes most, or how they interact, remains unknown.
- Coarse age-band resolution. Even the most granular cross-age-group studies identified (Section 1.2) compare broad bands (infant/child/adolescent/adult) rather than tracking continuous developmental trajectories; the field lacks data on how tryptophan-pathway metabolites change across the specific 0–2-year window when gut microbiome maturation, skin-barrier development, and AD onset are most dynamically co-occurring.
- No pediatric pharmacometabolomic studies. We identified no published pharmacometabolomic study reporting pediatric-stratified tryptophan-pathway outcomes for AD biologics or JAK inhibitors (Section 7): study [64] included a clinically characterized pediatric dupilumab subgroup, but whether its microbiome/tryptophan-metabolism findings were analyzed separately for that subgroup was not reported (Table 3); tralokinumab, lebrikizumab, and JAK inhibitors have no tryptophan-pathway outcome data identified in any age group.
- Confounder-adjusted data are absent. None of the studies identified here statistically account for feeding mode, antibiotic exposure, or dietary tryptophan intake when reporting pediatric tryptophan-pathway metabolite levels (Section 1.1), leaving open the possibility that reported “AD-associated” differences partly reflect unmeasured lifestyle or exposure confounding rather than AD pathophysiology itself.
- Cross-disease validation is absent. Whether a tryptophan-pathway biomarker signature is AD-specific, or shared across asthma, food allergy, chronic urticaria, and drug hypersensitivity, is a question we identified no study addressing (Section 10.1) and answerable with existing pediatric allergic-disease biobanks, without new specimen collection.
- No multi-omic integration model. We identified no published machine-learning or formal multi-omic integration model for tryptophan-pathway data in AD, at any age (Section 8); the component data types (metabolomics, cytokine panels, microbiome sequencing) exist, but not, to our knowledge, a framework that links them.
- Standardization is undeveloped. We identified no consensus reference ranges, preanalytical protocols, or interlaboratory quality standards for pediatric serum tryptophan-pathway metabolites (Section 10.2), a prerequisite for any of the above eventually translating into clinical use.
- Sex-stratification is unexamined. Whether tryptophan-pathway metabolism (kynurenine, serotonin/melatonin, or microbial indole branches) differs by sex in PAD has not been examined by any study identified here; given this review’s emphasis on age as a key stratification variable, sex represents a closely related and currently unaddressed dimension that future prospective pediatric cohorts should incorporate alongside age.

These gaps collectively define a research agenda rather than a single study: gaps 1, 2, and 8 and the causal-direction problem above are addressable by a single well-designed prospective pediatric cohort (the central research priority identified throughout this review); gaps 3 and 5 require access to existing clinical trial biobanks; gaps 6–7 are methodological/infrastructural rather than data-collection gaps and could, in principle, proceed in parallel with primary data collection. These gaps motivate the translational pipeline proposed in Section 10.

## 6. Tryptophan Metabolites as Candidate Biomarkers: A Proposed Framework

Adapting the standard framework used in contemporary AD biomarker research [11,19], tryptophan-pathway metabolites can be organized into four candidate biomarker categories. This categorization is conceptual, built by mapping the mechanistic evidence in Section 3 and Section 4 onto an established biomarker taxonomy; it is not yet an empirically validated classification for tryptophan metabolites specifically. No metabolite listed below has been assigned to its category on the basis of a validation study measuring the corresponding diagnostic or prognostic performance characteristic (e.g., sensitivity/specificity for diagnosis, correlation with a severity index, discriminative accuracy for endotype, or area-under-curve for treatment response prediction); the assignments instead reflect where each metabolite’s established mechanistic role (Section 3 and Section 4) would predict it to be most informative. This framework is therefore presented as a set of testable hypotheses for future biomarker-validation studies rather than as a summary of existing validation evidence.

Diagnostic biomarkers are candidates that could help distinguish AD from non-atopic skin inflammation or from other allergic conditions. Serum Kyn/Trp ratio and skin/serum indole-3-lactic acid are the most plausible candidates given their consistent involvement in barrier pathology [80,81], though neither has been formally validated for diagnostic discrimination in AD.

Severity biomarkers are candidates that track with disease activity. Neopterin/IDO activity [60] and the melatonin-responsive propionic acid axis [77] are the closest current analogs, though neither has been shown to correlate with a validated severity index such as SCORAD or EASI: [60] reports only a between-group difference (AD versus controls), which establishes association with AD status, not with disease severity, and [77]’s mechanistic link to melatonin’s clinical effect (Section 4.2) has likewise not been benchmarked against a validated severity score. A tryptophan-pathway severity marker in the SCORAD/EASI-correlated sense already established for TARC (Section 2) remains to be demonstrated.

Endotype biomarkers are candidates that could stratify patients by underlying immune polarization (Th2 vs. Th17/Th22-skewed), echoing the pediatric-specific endotype transcriptomic work of Section 1.1 and Section 10 [12,15,16,17]. Kynurenine-pathway activity’s mechanistic link to Th2/Th17 polarization makes it a rational, but currently untested, endotype candidate.

Predictive biomarkers are candidates forecasting treatment response before or early in therapy. This is the least developed category and is addressed directly in Section 7.

## 7. Tryptophan Metabolism and Response to Targeted Therapies

For the cytokine-targeted biologics and JAK inhibitors discussed below, this is the area with the sparsest direct evidence in the current literature; they are presented here as identifying candidate future biomarkers rather than as a synthesis of established findings, reflecting the field’s current trajectory without overclaiming existing evidence. Tapinarof, by contrast, is not a cytokine-targeted biologic but a direct AhR agonist and offers the most mature clinical validation of the AhR-axis argument developed throughout this review.

Tapinarof (topical AhR agonist). Tapinarof (benvitimod) is a non-steroidal topical AhR agonist already approved for AD in children as young as two years, providing the most direct pharmacological validation of the AhR-centered framework proposed in Section 3 [95]. This validates AhR as a druggable target in AD; it does not, by itself, validate tryptophan-pathway metabolites as biomarkers, since tapinarof trials to date have not measured tryptophan, kynurenine, or indole metabolite levels alongside clinical response. A 48-week phase 3 open-label extension (ADORING 3) demonstrated sustained skin clearance and an extended treatment-free interval in both adults and children with AD [96]. Mechanistically, tapinarof drives NRF2-dependent regulation of keratinocyte SEMA3A expression [97] and inhibits IL-4/IL-13-induced tight junction impairment through combined AhR/ARNT activation and STAT6 inhibition [98], directly reinforcing the AhR–barrier link that motivates this review’s precision-medicine framework (Section 4.3 and Section 6). Consistent with an on-target pharmacodynamic effect, low-AhR-ligand dietary conditions have been shown to modulate the skin barrier and immune response in murine AD models, further supporting ligand availability as a determinant of AhR-pathway activity in the skin [99]. No study to date has reported tryptophan- or kynurenine-pathway metabolite changes accompanying tapinarof treatment; pairing pharmacodynamic metabolomics with tapinarof trials is therefore a direct, low-barrier extension of the biomarker framework proposed in Section 6.

Dupilumab (anti-IL-4Rα). The only intervention in the current literature with direct, tryptophan-pathway-relevant outcome data: dupilumab therapy normalized gut microbiome dysbiosis and tryptophan metabolism in AD patients alongside clinical improvement [64]. This is the strongest available proof-of-concept that a targeted biologic’s clinical effect is accompanied by, and plausibly mediated in part through, restoration of tryptophan-pathway function, though causality (restoration driving improvement vs. improvement permitting microbiome/metabolic normalization) cannot be established from an observational design. This is pharmacodynamic evidence: a treatment-associated change; not predictive evidence: [64] did not assess whether pretreatment tryptophan-pathway status predicted subsequent clinical response.

Tralokinumab and lebrikizumab (anti-IL-13). No study in the current literature reports tryptophan-pathway or kynurenine/indole-metabolite outcomes for either agent. Given that IL-13 directly suppresses filaggrin and barrier-associated gene expression, the same downstream endpoint affected by AhR/indole signaling (Section 4.3), there is a mechanistic rationale to hypothesize that anti-IL-13 blockade could indirectly normalize AhR-pathway activity via barrier restoration, a rationale reinforced by direct evidence of AhR/IL-13 signaling crosstalk in human keratinocytes [100], but this remains an explicit evidence gap rather than a reported finding, and we identify it as a priority for future pharmacometabolomic sub-studies.

JAK inhibitors. Similarly absent from the current literature with respect to tryptophan-pathway outcomes. JAK–STAT signaling intersects the kynurenine pathway indirectly (IDO1 induction is IFN-γ/JAK-STAT1-dependent), providing mechanistic plausibility for a JAK-inhibitor effect on kynurenine flux, but again this is hypothesis-generating rather than evidenced.

Beyond tapinarof’s mechanistic AhR validation, this section identifies tryptophan/kynurenine/indole metabolites as candidate biomarkers of two distinct kinds: pharmacodynamic (tracking treatment-associated change, as in [64]) and, more speculatively, predictive (forecasting response from pretreatment values). Current evidence supports only the former; no study identified here has tested whether pretreatment tryptophan-pathway status predicts subsequent response to any cytokine-targeted biologic or JAK inhibitor, so the predictive category remains a testable hypothesis rather than an evidenced claim. (Table 3).

## 8. Integration with Multi-Omics

No single tryptophan metabolite is likely to function as a standalone clinical biomarker; the more defensible framing, consistent with the direction of the wider field, is that tryptophan-pathway metabolites gain diagnostic and predictive value when integrated with complementary omic layers:

- Metabolomics beyond tryptophan. Integrated plasma metabolomic and cytokine panels already demonstrate that a distinct immunometabolic signature, not a single analyte, best discriminates AD immune states [63], providing methodological precedent for a similar multi-analyte tryptophan-inclusive panel; that precedent, however, comes from an adult cohort (Table 3) and remains unvalidated in children.
- Cytokine co-profiling. Joint cytokine–metabolite panels are a natural next step, given the established Th2/Th17/Th22 cytokine biomarker landscape and the mechanistic link between kynurenine-pathway flux and Th2/Th17 polarization (Section 4.1); the pediatric transcriptomic endotype literature that already profiles cytokine signatures at the tissue level [15,16,17,18] provides implicit support for this direction.
- Microbiome sequencing. Pairing serum/skin metabolomics with 16S or shotgun metagenomic sequencing of the same patients is the most direct way to establish producer–metabolite–phenotype causal chains, since a substantial share of the mechanistic evidence in Section 4.3 concerns bacterially produced indole metabolites; this design has precedent in adult/preclinical AhR-microbiome studies [101], though not yet in a PAD cohort.
- Machine learning integration. Machine-learning multi-omic integration for PAD is not, in fact, unprecedented: Jiang et al. combined transcriptome and gut microbiota data in infants (mean age~11–17 months) to classify AD with F1 = 0.84 [102], and a 2026 preprint extends this approach to gene expression, cytokine, antibody, and environmental data in children aged 12–36 months [103], preprint, not yet peer-reviewed. Neither, however, incorporates targeted tryptophan-pathway metabolomics; we identify this specific extension, not the general ML-multi-omic approach, which is already established, as the explicit methodological gap and future direction.

Of these four directions, cytokine co-profiling and microbiome sequencing are the most immediately feasible in a pediatric cohort, since both build directly on sample types and infrastructure already used in the pediatric endotype and microbiome literature cited above; the multi-analyte metabolomic panel and machine-learning integration are better framed as second-stage goals, to be pursued once a dedicated pediatric tryptophan-pathway dataset, of the kind called for throughout Section 5, actually exists.

## 9. Discussion: Which Mechanisms Are Age-Invariant, and Which Remain Developmentally Unconfirmed?

The age-specific framing signaled in the title of this review is not a stylistic choice but its organizing argument. The pediatric evidence base for the tryptophan-metabolic pathway is sparse relative to the adult and preclinical literature from which much of its mechanistic plausibility is drawn (Section 1.4), and this evidence is qualitatively different in kind, not simply smaller in volume: pediatric skin and immune biology differ from adult biology in ways directly relevant to this pathway (Section 1.1, Section 1.2 and Section 1.3), so mechanistic relevance established in adults or in preclinical models cannot be assumed to transfer to children without direct pediatric validation. Every biomarker and mechanism discussed in this review is accordingly evaluated against that standard, and distinguished explicitly as pediatric-direct, adult-derived, or preclinical/mechanistic evidence (Section 1.4; Supplementary Table S1) wherever the underlying studies allow it.

The evidence assembled in Section 3, Section 4, Section 5, Section 6, Section 7 and Section 8 does not support a single, uniform answer to the question posed in the Abstract. Instead, it separates into two evidentiary tiers.

Plausibly age-invariant mechanisms. The core biochemical logic of the tryptophan–AhR–filaggrin axis (Section 3, point 3; Section 4.3) holds regardless of age, at least in principle: IDO1 induction by IFN-γ and Th2/Th17 cytokines is a general immunometabolic response documented across tissue types and species [39,41], and AhR’s role in keratinocyte differentiation and barrier gene expression is conserved from murine models to adult human skin to the limited pediatric transcriptomic data available [15,16,17,51]. Where pediatric transcriptomic studies exist, they show the same Th2/Th17/Th22-skewed inflammatory signature and barrier gene dysregulation reported in adults [15,16,17,18], consistent with, though not proof of, comparable upstream metabolic involvement. Microbial indole production is likewise mechanistically age-invariant at the level of the AhR-ligand interaction itself; what varies by age is not the mechanism but the input, since gut and skin microbiome composition, and therefore indole output, changes substantially over the first years of life (Section 1.1).

Developmentally unconfirmed or plausibly age-specific candidates. In contrast, no pediatric reference ranges exist for serum Kyn/Trp ratio or indole metabolite concentrations, and the confounders identified in Section 1.1 (feeding mode, antibiotic exposure, microbiome maturation, dietary intake) affect these measurements in ways that are themselves age-dependent, meaning an identical serum value could carry different biological meaning in an infant versus an adolescent. The two direct pediatric signals identified in this review [60], showing elevated kynurenine-pathway activity in an AD-specific subgroup, and [26], the first study to link fecal tryptophan/indole trajectories to eczema onset, demonstrate that pediatric-specific tryptophan-pathway differences are measurable, but neither was designed to establish reference ranges, and both used the disease designation of their underlying cohort (broader allergic disease; doctor-diagnosed eczema) rather than formal Hanifin–Rajka diagnostic criteria and standardized SCORAD severity scoring. The melatonin pathway presents a distinct pattern: its single strongest piece of clinical evidence is a pediatric RCT [75], but this may reflect the age-skewed prominence of sleep disturbance as an AD comorbidity in children rather than an age-invariant biological effect, and no comparable pediatric trial exists for the serotonin sub-pathway, whose evidence base (Section 4.2) is almost entirely adult-derived [67,68,69,70,71].

Taken together, this asymmetry is itself a central finding of this review: the mechanistic architecture of the tryptophan–AhR axis appears general across pediatric and adult skin alike, yet essentially none of its quantitative, diagnostic, or age-specific clinical parameters have been established in children. Extending adult-derived cutoffs or biomarker thresholds to pediatric populations without independent validation would therefore be premature, and this distinction should guide how the biomarker framework proposed in Section 6 is tested going forward.

For completeness, we note that age-related immunological divergence in AD is not confined to the pediatric–adult transition: AD in older adults shows an inverse cytokine pattern relative to healthy skin aging, with Th2/Th22-associated cytokines declining rather than rising, Th1/Th17 activation increasing, and terminal differentiation markers (filaggrin, loricrin) increasing [104]. No study identified here reports tryptophan-, kynurenine-, or indole-pathway metabolites in geriatric AD specifically. This end of the age spectrum falls outside this review’s pediatric focus and is flagged here only as an open question for future work.

## 10. Future Research Directions and Translational Perspectives

Synthesizing Section 1, Section 2, Section 3, Section 4, Section 5, Section 6, Section 7, Section 8 and Section 9, this review proposes the following conceptual pipeline as a roadmap for translating tryptophan metabolism into pediatric precision dermatology:

1. Clinical phenotype (age of onset, morphology, atopic comorbidity) is documented at presentation.
2. Cytokine/endotype profiling, building on established pediatric transcriptomic work [15,16,17,18] and serum endotype confirmation approaches [12], stratifies patients by dominant Th2/Th17/Th22 polarization.
3. Tryptophan-pathway metabolite profiling (serum Kyn/Trp ratio, targeted indole panel) adds a mechanistic readout of the barrier–microbiome axis not captured by cytokine profiling alone (Section 3 and Section 4).
4. Endotype refinement combines both layers into a composite endotype spanning immunological and metabolic/microbiome dimensions.
5. Therapy selection matches the refined endotype to the most mechanistically appropriate intervention: anti-IL-4Rα/anti-IL-13 biologics for Th2-dominant endotypes with what we term, for the purposes of this hypothetical framework, “preserved AhR-axis function”; AhR-agonist therapy (tapinarof) or indole/microbiome-directed interventions for endotypes with a corresponding hypothetical “barrier–AhR deficit”; melatonin as a low-risk adjunct where the serotonin–melatonin axis and sleep disturbance are prominent. These AhR-axis phenotype labels are proposed constructs for this conceptual pipeline, not validated clinical definitions: no diagnostic threshold, assay, or cutoff currently distinguishes “preserved” from “deficit” status in any patient, and their clinical use would require prospective definition and validation before they could guide therapy selection. (Section 5).

### 10.1. Relevance Beyond Atopic Dermatitis

Although this review is scoped to AD, the kynurenine pathway’s role in immune regulation is not disease-specific: it has been proposed as a broader link between allergic disease and the gut microbiome [105], with plausibility extending to other Th2-mediated conditions, asthma, food allergy, chronic urticaria, that share overlapping Th2/Th17 circuitry and, for food allergy and asthma, comparable microbiome dependence Within the AD-focused search strategy described in Section 1.4, no study reports tryptophan-pathway metabolites in pediatric asthma, food allergy, chronic urticaria, or drug hypersensitivity. That search was not designed as a dedicated cross-disease search of these four conditions, so this observation should be read as an apparent gap consistent with the present search rather than a confirmed absence of evidence; verifying it would require a purpose-built search of each condition. A tryptophan-pathway biomarker panel validated in PAD (Section 6) would be a natural candidate for testing across this broader spectrum.

### 10.2. Translational and Implementation Barriers

The precision-medicine pipeline proposed above (and the biomarker categories in Section 6) will not reach clinical practice on mechanistic plausibility alone. Several concrete implementation barriers should be acknowledged directly:

- Cost and accessibility. Targeted LC-MS/MS metabolomic panels and single-cell multi-omic profiling (Section 1.1) are substantially more expensive and less accessible than existing serum biomarkers (Section 2), a gap that matters more in resource-constrained pediatric care settings.
- Standardization. No consensus reference ranges, preanalytical protocols, or interlaboratory quality-control standards yet exist for pediatric serum tryptophan-pathway metabolites, unlike the mature standardization around TARC and specific IgE testing.
- Turnaround time versus clinical decision speed. Metabolomic and single-cell assays take days to weeks, while treatment decisions in symptomatic PAD are often made at the point of care; a panel that cannot return results in that window will struggle for adoption.
- Regulatory validation. None of the candidate biomarkers in Section 6 have undergone the analytical and clinical validation (e.g., IVDR in the EU, FDA clearance in the US) required for routine use.

These barriers do not diminish the scientific rationale developed in Section 3, Section 4, Section 5, Section 6, Section 7, Section 8 and Section 9; they define the translational work required once that rationale is confirmed, not a reason to doubt it.

## 11. Conclusions

Two distinct bodies of evidence underlie this review, at very different stages of maturity. The biological mechanism—AhR-dependent regulation of keratinocyte differentiation and skin-barrier integrity—is well established, largely in adult and preclinical systems, and is already therapeutically targetable. Tryptophan-pathway metabolites as PAD biomarkers, by contrast, remain a candidate framework rather than a validated one: within the literature search described, only two studies directly linked the tryptophan pathway to PAD [26,60], and both have important limitations, including small sample sizes, the use of study-specific eczema or allergic-disease criteria rather than formal Hanifin–Rajka diagnostic criteria/standardized SCORAD severity scoring, and the absence of pediatric reference ranges. The strength of the mechanistic evidence should not be read as strength of the biomarker evidence; the two require separate validation.

The most important translational priority is therefore not to propose a new mechanism, but to validate the existing one in adequately powered prospective pediatric cohorts. Longitudinal profiling of fecal tryptophan and indole metabolites, combined with gut microbiome sequencing, standardized clinical assessment of AD severity, skin-barrier measurements, and cytokine profiling, would provide the integrated evidence currently missing. Particular attention should be given to the independent validation of indole-3-lactic acid as a potential biomarker and to determining whether metabolite profiles differ across pediatric age groups and disease states.

Before these biomarkers can be considered for clinical application, pediatric-specific preanalytical protocols and age-stratified reference ranges must be established. A standardized, longitudinal multi-omic approach would therefore represent the most direct strategy to determine whether the tryptophan–indole–AhR axis can be translated from a biologically plausible mechanism into a clinically useful biomarker or therapeutic framework for PAD.

## Figures and Tables

**Figure 1:**
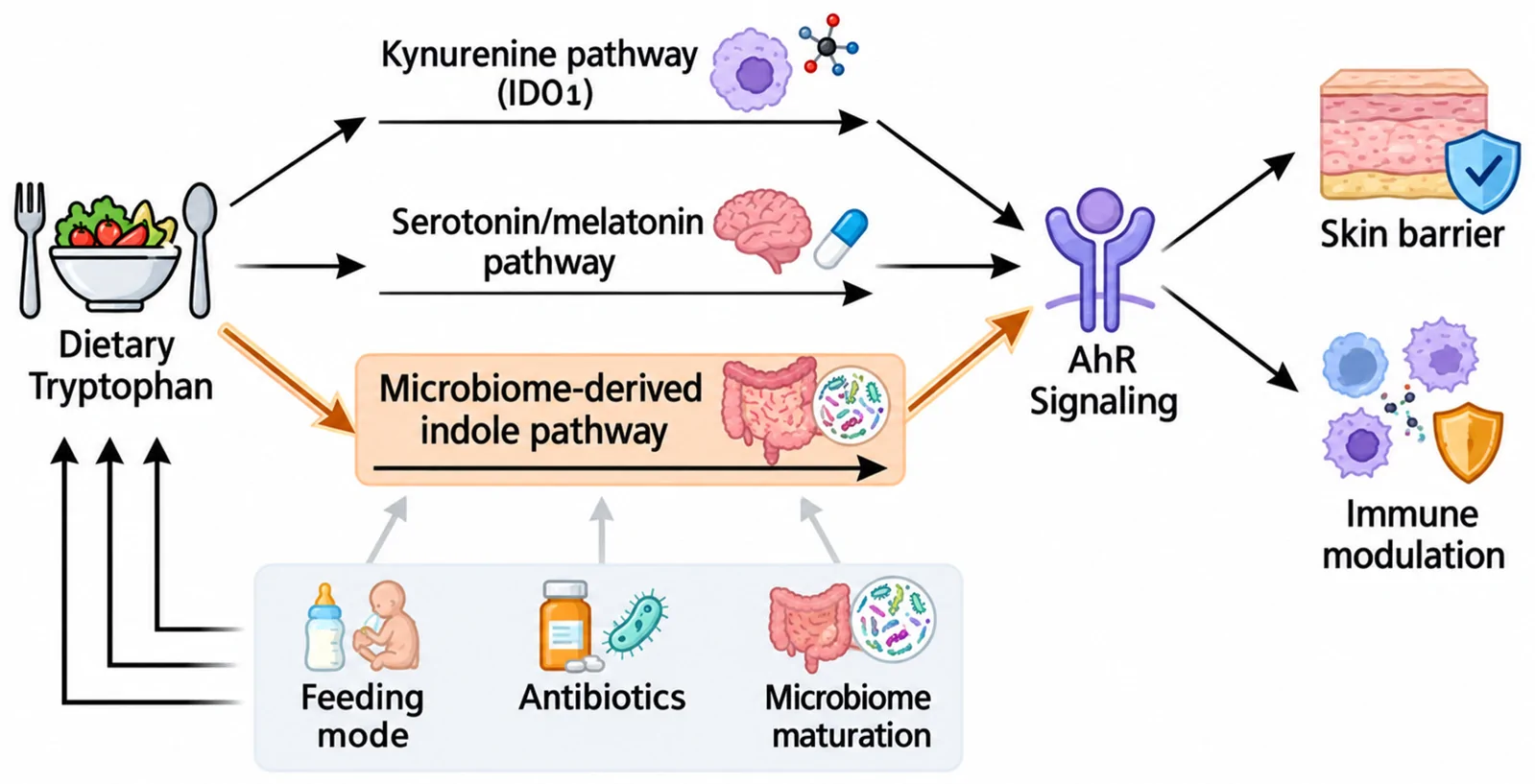
Schematic overview of the proposed interplay between tryptophan-metabolic pathways, the gut–skin axis, and atopic dermatitis, highlighting potential mechanisms involving the kynurenine, serotonin, and indole pathways and AhR signaling.

**Table 1:** Age bands used to characterize developmental differences relevant to atopic dermatitis and tryptophan metabolism. These age bands were developed specifically for this review, drawing on developmental transition points relevant to skin-barrier and microbiome maturation (weaning) and pubertal onset, rather than adopted from a single established pediatric staging system; age ranges are defined as left-closed, right-open intervals to ensure mutually exclusive categories.

Age Band	Age Range	Rationale
Infant	0 to <2 years	Skin barrier and gut microbiome are still undergoing maturation and are strongly influenced by feeding mode (breastfeeding versus formula feeding) and mode of delivery.
Child	2 to <12 years	The gut microbiome is largely stabilized after weaning, while immune and hormonal profiles remain predominantly pre-pubertal.
Adolescent	12 to <18 years	Pubertal hormonal changes occur during this period, accompanied by progressive transition of the immune profile toward the adult pattern.
Adult	≥18 years	Serves as a reference population for a substantial proportion of the mechanistic atopic dermatitis literature and represents a relatively stable physiological state compared with infancy, childhood, and adolescence, during which microbiome, immune, and hormonal development may substantially influence atopic dermatitis-related mechanisms.

**Table 2:** Key studies supporting age-dependent immunological and molecular heterogeneity in atopic dermatitis, with relevance to tryptophan-pathway signaling.

Ref.	Study	Population/ Age Range	Method	Key Age-Related Finding/Relevance	Relevance to Tryptophan Metabolism
[15]	Esaki et al., 2016	Children with early-onset AD (<5 years) and healthy controls	Skin transcriptomics	Early-onset PAD was predominantly Th2-polarized but also exhibited prominent Th17-associated inflammation.	Establishes an early-onset Th2/Th17 inflammatory context that may influence downstream IDO1-kynurenine signaling.
[16]	Brunner et al., 2018	Children with early-onset AD and healthy controls	Skin transcriptomics and lipidomics	PAD was characterized by Th2/Th17/Th22-centered inflammation accompanied by alterations in lipid metabolism and skin-barrier pathways.	Indicates that inflammatory and barrier-associated metabolic alterations may interact with tryptophan-pathway signaling.
[17]	Renert-Yuval et al., 2021	Infants (0–5 years), children (6–11 years), adolescents (12–17 years), and adults (≥18 years)	Skin RNA sequencing, RT-PCR, and immunohistochemistry	A shared Th2/Th22 inflammatory signature was observed across age groups, with distinct age-specific differences in Th17, Th1, cytokine, and skin-barrier profiles.	Provides strong evidence that immune and barrier programs potentially relevant to metabolic signaling vary across developmental stages.
[18]	Czarnowicki et al., 2020	Patients with AD ranging from infancy to adulthood	Circulating T-cell subset immunophenotyping	Age-related shifts in pathogenic circulating T-cell subsets were identified, complementing molecular profiling of lesional skin.	Supports the presence of age-dependent systemic immune differences that could influence circulating tryptophan metabolites.
[24]	Baldonado et al., 2026	Pediatric patients (0–17 years), adults (18–59 years), and older adults (≥60 years) with AD	Single-cell multi-omics of peripheral blood mononuclear cells	High-resolution peripheral immune profiling revealed age-associated differences in transcriptomic and surface-protein profiles across developmental stages.	Provides peripheral single-cell evidence for age-dependent immune programs potentially upstream of tryptophan-pathway regulation; targeted tryptophan-pathway metabolomics was not performed.

AD, atopic dermatitis; IDO1, indoleamine 2,3-dioxygenase 1; RT-PCR, reverse transcription polymerase chain reaction.

**Table 3:** Studies screened for tryptophan/kynurenine-pathway relevance in PAD, ordered by evidentiary status: confirmed PAD-specific signals, near-pediatric comparators of uncertain tryptophan-pathway relevance, and comparator studies excluded or provisionally excluded from the pediatric-signal count.

Ref.	Study	Population	Study Design	Analytical Approach	Key Findings Relevant to Tryptophan Metabolism	Evidence Status
[61]	Huang et al., 2014	Children with AD and healthy controls	Case–control, cross-sectional	Serum UHPLC–MS metabolomics with targeted eicosanoid analysis	AD was associated with alterations in eicosanoids, bile acids, carnitines, fatty acids, and other metabolites. A dedicated analysis of the tryptophan/kynurenine pathway was not reported.	Pediatric comparator; broad metabolomic evidence without a confirmed tryptophan-pathway signal
[60]	Ünüvar et al., 2019	Children aged 4 months–17 years (*n* = 205 overall; *n* = 25 with AD)	Cross-sectional	Serum neopterin immunoassay and HPLC quantification of tryptophan and kynurenine; IDO1 activity calculated from the kynurenine/tryptophan ratio	Children with AD had higher serum neopterin, tryptophan, kynurenine, and calculated IDO1 activity than controls (*p* < 0.05), indicating altered kynurenine-pathway activity.	Direct pediatric evidence of a tryptophan/kynurenine-pathway signal; AD subgroup derived from a broader allergic-disease cohort
[62]	Kim et al., 2023	Children with AD (*n* = 17) and healthy controls (*n* = 24)	Cross-sectional	Plasma metabolomics by UPLC–QTOF–MS	Five differential metabolites, all lysophosphatidylcholines, were decreased in children with AD. Tryptophan metabolism was not the primary focus, and no direct tryptophan-pathway alteration was established.	Pediatric comparator; lipid-metabolomic evidence without a confirmed tryptophan-pathway signal
[63]	Ma et al., 2024	Adults with AD (*n* = 20) and healthy controls (*n* = 24)	Cross-sectional	Untargeted plasma metabolomics by UPLC–Q Exactive Orbitrap MS with cytokine profiling	Tryptophan and indole-3-acrylic acid were significantly decreased in AD, supporting altered tryptophan/indole metabolism.	Adult comparator; direct tryptophan-pathway signal; not pediatric evidence
[64]	Yang et al., 2024	Pediatric and adult participants; pediatric subgroup included 15 patients with AD aged < 18 years and 18 healthy controls within the overall cohort (44 AD and 48 healthy controls)	Interventional, pre–post dupilumab study	Gut microbiome profiling and serum metabolomics	Dupilumab treatment was associated with changes in gut microbiome composition and tryptophan/indole metabolism, supporting a potential microbiome–tryptophan-metabolic link in AD.	Pediatric subgroup clinically characterized at baseline; whether the microbiome and tryptophan-metabolism findings were separately analyzed in the pediatric subgroup remains unclear
[65]	Yu et al., 2025	Preschool children with AD (*n* = 23) and healthy controls (*n* = 23)	Cross-sectional	Untargeted serum metabolomics by UPLC–QTOF–MS	Multiple metabolites differed between groups, with enrichment of the phenylalanine metabolism pathway. No specific alteration in the tryptophan/kynurenine pathway was established.	Pediatric comparator; metabolomic evidence without a confirmed tryptophan-pathway signal
[26]	Wieser et al., 2026	Infants (prospective cohort, *n* = 40; eczema subgroups *n* = 7–12)	Prospective longitudinal cohort	Fecal metabolomics (tryptophan, tryptamine, indole-3-lactic acid, and tryptophan/indole ratios)	Fecal tryptophan and tryptamine elevated in early-onset eczema by 6 months of age; indole-3-lactic acid reduced ahead of late-onset eczema diagnosis; several tryptophan/indole ratios reached AUC > 0.8 for discriminating eczema trajectory.	Direct pediatric evidence of a tryptophan/indole-pathway signal; the only longitudinal study identified in this table, though with small eczema subgroups (*n* = 7–12) and a purely observational design

AD, atopic dermatitis; AUC, area under the receiver operating characteristic curve; HPLC, high-performance liquid chromatography; IDO1, indoleamine 2,3-dioxygenase 1; liquid chromatography–mass spectrometry; MS, mass spectrometry; UPLC–QTOF–MS, ultra-performance liquid chromatography–quadrupole time-of-flight mass spectrometry; UHPLC–MS, ultra-high-performance liquid chromatography–mass spectrometry.

## Data Availability

No new empirical data were created or analyzed in this study. This review is based exclusively on previously published scientific literature and publicly available data reported in the cited studies. Data sharing is not applicable to this article.

## Supplementary Materials

The following supporting information can be downloaded at: https://www.mdpi.com/article/10.3390/biomedicines14102140/s1, Table S1. Classification of reviewed literature.

## Abbreviations

16S rRNA	16S ribosomal RNA
AD	atopic dermatitis
AhR	aryl hydrocarbon receptor
ARNT	aryl hydrocarbon receptor nuclear translocator
AUC	area under the receiver operating characteristic curve
HPLC	high-performance liquid chromatography
IDO1	indoleamine 2,3-dioxygenase 1
IFN-γ	interferon-gamma
IL-4	interleukin-4
IL-4Rα	interleukin-4 receptor alpha
IL-13	interleukin-13
ILA	indole-3-lactic acid
JAK	Janus kinase
JAK–STAT	Janus kinase–signal transducer and activator of transcription
Kyn	kynurenine
Kyn/Trp	kynurenine-to-tryptophan ratio
LC–MS	liquid chromatography–mass spectrometry
MS	mass spectrometry
NRF2	nuclear factor erythroid 2-related factor 2
PAD	pediatric atopic dermatitis
PBMCs	peripheral blood mononuclear cells
RCT	randomized controlled trial
ROC	receiver operating characteristic
RT-PCR	reverse transcription polymerase chain reaction
SCORAD	Scoring Atopic Dermatitis
STAT1	signal transducer and activator of transcription 1
STAT6	signal transducer and activator of transcription 6
TDO	tryptophan 2,3-dioxygenase
Th2	T helper 2
Th17	T helper 17
Th22	T helper 22
Trp	tryptophan
TSLP	thymic stromal lymphopoietin
UHPLC–MS	ultra-high-performance liquid chromatography–mass spectrometry
UPLC–QTOF–MS	ultra-performance liquid chromatography–quadrupole time-of-flight mass spectrometry

## References

[1] Artusa S., Mazzuca G., Piacentini G., Castagnoli R., Marseglia G.L., Pietrobelli A., Pecoraro L. (2024). Paediatric Atopic Dermatitis: The Unexpected Impact on Life with a Specific Look at the Molecular Level. Int. J. Mol. Sci..

[2] Cao X., Wang M., Zhou M., Mi Y., Guo Q., Fan Y., Guo Y. (2024). Global, Regional and National Burden of Paediatric Atopic Dermatitis: A Trend and Geographic Inequalities Analysis. Clin. Exp. Allergy.

[3] Ab Hadi H., Tarmizi A.I., Khalid K.A., Gajdács M., Aslam A., Jamshed S. (2021). The Epidemiology and Global Burden of Atopic Dermatitis: A Narrative Review. Life.

[4] Migliavaca C.B., Lazzarini R., Stein C., Escher G.N., de Gaspari C.N., dos Santos H.W.G., Myers D.E., Lemeshow A.R., Pinheiro M.R.T., Falavigna M. (2025). Prevalence of Atopic Dermatitis: A Systematic Review and Meta-Analysis. Dermatitis.

[5] Jin L., Ge J., Cheng Y., Deng D., Wan P. (2025). Worldwide Prevalence of Atopic Dermatitis in Children between 2000 and 2021. Ann. Allergy Asthma Immunol..

[6] Weidinger S., Simpson E.L., Silverberg J.I., Barbarot S., Eckert L., Mina-Osorio P., Rossi A.B., Brignoli L., Mnif T., Guillemin I. (2024). Burden of Atopic Dermatitis in Paediatric Patients: An International Cross-Sectional Study. Br. J. Dermatol..

[7] Silverberg J.I., Barbarot S., Gadkari A., Simpson E.L., Weidinger S., Mina-Osorio P., Rossi A.B., Brignoli L., Saba G., Guillemin I. (2021). Atopic Dermatitis in the Pediatric Population: A Cross-Sectional, International Epidemiologic Study. Ann. Allergy Asthma Immunol..

[8] Puerta Durango K., Chiesa Fuxench Z.C. (2024). Global Burden of Atopic Dermatitis: Examining Disease Prevalence Across Pediatric and Adult Populations World-Wide. Dermatol. Clin..

[9] Suárez-Fariñas M., Dhingra N., Gittler J., Shemer A., Cardinale I., de Guzman Strong C., Krueger J.G., Guttman-Yassky E. (2013). Intrinsic Atopic Dermatitis Shows Similar TH2 and Higher TH17 Immune Activation Compared with Extrinsic Atopic Dermatitis. J. Allergy Clin. Immunol..

[10] Tokura Y., Hayano S. (2022). Subtypes of Atopic Dermatitis: From Phenotype to Endotype. Allergol. Int..

[11] Park C.O., Kim S.M., Lee K.H., Bieber T. (2024). Biomarkers for Phenotype-Endotype Relationship in Atopic Dermatitis: A Critical Review. eBioMedicine.

[12] Bakker D.S., Nierkens S., Knol E.F., Giovannone B., Delemarre E.M., van der Schaft J., van Wijk F., de Bruin-Weller M.S., Drylewicz J., Thijs J.L. (2021). Confirmation of Multiple Endotypes in Atopic Dermatitis Based on Serum Biomarkers. J. Allergy Clin. Immunol..

[13] Chiricozzi A., Levi A., Palladino C., Girolomoni G. (2025). Enabling Precision Medicine with Biomarkers of Response to Treatment in Atopic Dermatitis: Where Are We Now? A Narrative Review. Front. Med..

[14] Fonacier L., Mawhirt S., Stern H., Roellke E., Singer S., Hunt A., Lio P. (2026). Precision Medicine in Atopic Dermatitis: Present and Future. Ann. Allergy Asthma Immunol..

[15] Esaki H., Brunner P.M., Renert-Yuval Y., Czarnowicki T., Huynh T., Tran G., Lyon S., Rodriguez G., Immaneni S., Johnson D.B. (2016). Early-Onset Pediatric Atopic Dermatitis Is TH2 but Also TH17 Polarized in Skin. J. Allergy Clin. Immunol..

[16] Brunner P.M., Israel A., Zhang N., Leonard A., Wen H.-C., Huynh T., Tran G., Lyon S., Rodriguez G., Immaneni S. (2018). Early-Onset Pediatric Atopic Dermatitis Is Characterized by TH2/TH17/TH22-Centered Inflammation and Lipid Alterations. J. Allergy Clin. Immunol..

[17] Renert-Yuval Y., Del Duca E., Pavel A.B., Fang M., Lefferdink R., Wu J., Diaz A., Estrada Y.D., Canter T., Zhang N. (2021). The Molecular Features of Normal and Atopic Dermatitis Skin in Infants, Children, Adolescents, and Adults. J. Allergy Clin. Immunol..

[18] Czarnowicki T., He H., Canter T., Han J., Lefferdink R., Erickson T., Rangel S.M., Kameyama N., Kim H.J., Pavel A.B. (2020). Evolution of Pathologic T-Cell Subsets in Patients with Atopic Dermatitis from Infancy to Adulthood. J. Allergy Clin. Immunol..

[19] Renert-Yuval Y., Thyssen J.P., Bissonnette R., Bieber T., Kabashima K., Hijnen D., Guttman-Yassky E. (2021). Biomarkers in Atopic Dermatitis—A Review on Behalf of the International Eczema Council. J. Allergy Clin. Immunol..

[20] Tan Y., Peng G., Abudouwanli A., Zhao W., Sun Q., Yang M., Ogawa H., Okumura K., Niyonsaba F. (2026). Tryptophan Metabolism at the Crossroads of Immunity, Barrier Function, and the Microbiome in Atopic Dermatitis. Clin. Rev. Allergy Immunol..

[21] Stefanovic N., Irvine A.D. (2024). Filaggrin and Beyond: New Insights into the Skin Barrier in Atopic Dermatitis and Allergic Diseases, From Genetics to Therapeutic Perspectives. Ann. Allergy Asthma Immunol..

[22] Virolainen S.J., Satish L., Biagini J.M., Chaib H., Chang W.C., Dexheimer P.J., Dixon M.R., Dunn K., Fletcher D., Forney C. (2024). Filaggrin Loss-of-Function Variants Are Associated with Atopic Dermatitis Phenotypes in a Diverse, Early-Life Prospective Cohort. JCI Insight.

[23] Hoyer A., Rehbinder E.M., Färdig M., Asad S., Lødrup Carlsen K.C., Endre K.M.A., Granum B., Haugen G., Hedlin G., Monceyron Jonassen C. (2022). Filaggrin Mutations in Relation to Skin Barrier and Atopic Dermatitis in Early Infancy. Br. J. Dermatol..

[24] Baldonado G.C.L., Kumar S., Jin J., Fang X., Ildardashty A., Braun M., Neuhaus I.M., Mathes E., Bhutani T., Liao W. (2026). Immunological Differences in Atopic Dermatitis Across Age Groups: Insights from Single-Cell Multi-Omics. J. Transl. Med..

[25] Hoskinson C., Medeleanu M.V., Reyna M.E., Dai D.L.Y., Chowdhury B., Moraes T.J., Mandhane P.J., Simons E., Kozyrskyj A.L., Azad M.B. (2024). Antibiotics Taken within the First Year of Life Are Linked to Infant Gut Microbiome Disruption and Elevated Atopic Dermatitis Risk. J. Allergy Clin. Immunol..

[26] Wieser N.V., Admiraal I., Weiss R., Ghiboub M., Davids M., Lefèvre A., Emond P., Powell E.A., Sim K., Kroll J.S. (2026). Fecal Tryptophan Metabolism Predicts the Development of Infant Eczema: A Prospective Longitudinal Study. Gut Microbes.

[27] Prescott S.M., Billeci N., Gotcher M., Patel S., Almon A., Morgan H., Abukhalaf D., Groer M. (2025). Tryptophan as a Biomarker of Pregnancy-Related Immune Expression and Modulation. Front. Reprod. Health.

[28] El-Heis S., Crozier S.R., Robinson S.M., Harvey N.C., Cooper C., Inskip H.M., Godfrey K.M., Southampton Women’s Survey Study Group (2016). Higher Maternal Serum Nicotinamide/Related Metabolites in Late Pregnancy Associated with Lower Risk of Offspring Atopic Eczema at 12 Months. Clin. Exp. Allergy.

[29] Fujisawa T., Nagao M., Hiraguchi Y., Katsumata H., Nishimori H., Iguchi K., Kato Y., Higashiura M., Ogawauchi I., Tamaki K. (2009). Serum TARC/CCL17 in Children with Atopic Dermatitis: Elevated Normal Levels in Infancy, Age-Specific Analysis. Pediatr. Allergy Immunol..

[30] Yamada Y., Yoshihara S. (2026). Serum TARC Levels Reflect the Severity and Prognosis of Atopic Dermatitis in Infants Under 6 Months of Age: A Retrospective Study in a Pediatric Primary Care Practice. J. Dermatol..

[31] Ahrens B., Schulz G., Bellach J., Niggemann B., Beyer K. (2015). Chemokine Levels in Serum of Children with Atopic Dermatitis with Regard to Severity and Sensitization Status. Pediatr. Allergy Immunol..

[32] Gołuchowska N., Ząber A., Walczewska S., Będzichowska A., Brodaczewska K., Majewska A., Kalicki B., Tomaszewska A. (2025). Elevated Serum Levels of miRNA-155 in Children with Atopic Dermatitis: A Potential Biomarker of Disease. Int. J. Mol. Sci..

[33] Lv Y., Qi R., Xu J., Di Z., Zheng H., Huo W., Zhang L., Chen H., Gao X. (2014). Profiling of Serum and Urinary microRNAs in Children with Atopic Dermatitis. PLoS ONE.

[34] Geng R.S.Q., Buhler K.A., Choi M.Y., Croitoru D., Pope E., Fritzler M.J., Sibbald C. (2024). Serum Th1, Th2, Th17, and Innate Immune System Biomarkers Are Elevated in Pediatric Alopecia Areata With and Without Concurrent Atopic Dermatitis: A Cross-Sectional Study. JAAD Int..

[35] McAleer M.A., Jakasa I., Hurault G., Sarvari P., McLean W.H.I., Tanaka R.J., Kezic S., Irvine A.D. (2019). Systemic and Stratum Corneum Biomarkers of Severity in Infant Atopic Dermatitis Include Markers of Innate and T Helper Cell-Related Immunity and Angiogenesis. Br. J. Dermatol..

[36] Comai S., Bertazzo A., Brughera M., Crotti S. (2020). Tryptophan in Health and Disease. Adv. Clin. Chem..

[37] Holeček M. (2026). Serotonin, Kynurenine, and Indole Pathways of Tryptophan Metabolism in Humans in Health and Disease. Nutrients.

[38] Xue C., Li G., Zheng Q., Gu X., Shi Q., Su Y., Chu Q., Yuan X., Bao Z., Lu J. (2023). Tryptophan Metabolism in Health and Disease. Cell Metab..

[39] Miyamoto K., Sujino T., Kanai T. (2024). The Tryptophan Metabolic Pathway of the Microbiome and Host Cells in Health and Disease. Int. Immunol..

[40] Zhang J., Liu Y., Zhi X., Xu L., Tao J., Cui D., Liu T.F. (2024). Tryptophan Catabolism via the Kynurenine Pathway Regulates Infection and Inflammation. Inflamm. Res..

[41] Pallotta M.T., Rossini S., Suvieri C., Coletti A., Orabona C., Macchiarulo A., Volpi C., Grohmann U. (2022). Indoleamine 2,3-Dioxygenase 1 (IDO1): Up-to-Date Overview of an Eclectic Immunoregulatory Enzyme. FEBS J..

[42] Seo S.K., Kwon B. (2023). Immune Regulation through Tryptophan Metabolism. Exp. Mol. Med..

[43] Yu J., Luo Y., Zhu Z., Zhou Y., Sun L., Gao J., Sun J., Wang G., Yao X., Li W. (2019). A Tryptophan Metabolite of the Skin Microbiota Attenuates Inflammation in Atopic Dermatitis through the Aryl Hydrocarbon Receptor. J. Allergy Clin. Immunol..

[44] Zelante T., Iannitti R.G., Cunha C., De Luca A., Giovannini G., Pieraccini G., Zecchi R., D’Angelo C., Massi-Benedetti C., Fallarino F. (2013). Tryptophan Catabolites from Microbiota Engage Aryl Hydrocarbon Receptor and Balance Mucosal Reactivity via Interleukin-22. Immunity.

[45] Fang Z., Pan T., Li L., Wang H., Zhu J., Zhang H., Zhao J., Chen W., Lu W. (2022). Bifidobacterium longum Mediated Tryptophan Metabolism to Improve Atopic Dermatitis via the Gut–Skin Axis. Gut Microbes.

[46] Ye X., Li H., Anjum K., Zhong X., Miao S., Zheng G., Liu W., Li L. (2022). Dual Role of Indoles Derived from Intestinal Microbiota on Human Health. Front. Immunol..

[47] De Juan A., Segura E. (2021). Modulation of Immune Responses by Nutritional Ligands of Aryl Hydrocarbon Receptor. Front. Immunol..

[48] Dawe H.R., Di Meglio P. (2025). The Aryl Hydrocarbon Receptor (AHR): Peacekeeper of the Skin. Int. J. Mol. Sci..

[49] Furue M., Hashimoto-Hachiya A., Tsuji G. (2019). Aryl Hydrocarbon Receptor in Atopic Dermatitis and Psoriasis. Int. J. Mol. Sci..

[50] Uberoi A., Bartow-McKenney C., Zheng Q., Flowers L., Campbell A., Knight S.A.B., Chan N., Wei M., Lovins V., Bugayev J. (2021). Commensal Microbiota Regulates Skin Barrier Function and Repair via Aryl Hydrocarbon Receptor Signaling. Cell Host Microbe.

[51] Haas K., Weighardt H., Deenen R., Köhrer K., Clausen B., Zahner S., Boukamp P., Bloch W., Krutmann J., Esser C. (2016). Aryl Hydrocarbon Receptor in Keratinocytes Is Essential for Murine Skin Barrier Integrity. J. Investig. Dermatol..

[52] Polonio C.M., McHale K.A., Sherr D.H., Rubenstein D., Quintana F.J. (2025). The Aryl Hydrocarbon Receptor: A Rehabilitated Target for Therapeutic Immune Modulation. Nat. Rev. Drug Discov..

[53] Lee J., Choi H.-K., Shin H.S., Kim G.-D. (2024). Natural Products as Modulators of Aryl Hydrocarbon Receptor Signaling in Atopic Dermatitis Management. Molecules.

[54] Wang Y., Zhang Y., Wang W., Zhang Y., Dong X., Liu Y. (2025). Diverse Physiological Roles of Kynurenine Pathway Metabolites. Metabolites.

[55] Tanaka M., Tóth F., Polyák H., Szabó Á., Mándi Y., Vécsei L. (2021). Immune Influencers in Action: Metabolites and Enzymes of the Tryptophan-Kynurenine Metabolic Pathway. Biomedicines.

[56] Klaessens S., Stroobant V., De Plaen E., Van den Eynde B.J. (2022). Systemic Tryptophan Homeostasis. Front. Mol. Biosci..

[57] Grishanova A.Y., Perepechaeva M.L. (2024). Kynurenic Acid/AhR Signaling at the Junction of Inflammation and Cardiovascular Diseases. Int. J. Mol. Sci..

[58] Manni G., Mondanelli G., Scalisi G., Pallotta M.T., Nardi D., Padiglioni E., Romani R., Talesa V.N., Puccetti P., Fallarino F. (2020). Pharmacologic Induction of Endotoxin Tolerance in Dendritic Cells by L-Kynurenine. Front. Immunol..

[59] Harden J.L., Lewis S.M., Lish S.R., Suárez-Fariñas M., Gareau D., Lentini T., Johnson-Huang L.M., Krueger J.G., Lowes M.A. (2016). The Tryptophan Metabolism Enzyme L-Kynureninase Is a Novel Inflammatory Factor in Psoriasis and Other Inflammatory Diseases. J. Allergy Clin. Immunol..

[60] Ünüvar S., Erge D., Kılıçarslan B., Gözükara Bağ H.G., Çatal F., Girgin G., Baydar T. (2019). Neopterin Levels and IDO Activity as Biomarkers of Immune System Activation and Childhood Allergic Diseases. Ann. Lab. Med..

[61] Huang Y., Chen G., Liu X., Shao Y., Gao P., Xin C., Cui Z., Zhao X., Xu G. (2014). Serum Metabolomics Study and Eicosanoid Analysis of Childhood Atopic Dermatitis Based on LC-MS. J. Proteome Res..

[62] Kim J., Park Y.M., Lee S.Y., Kim B.S., Lee Y.K., Lee D.W., Nam M.H., Hong S.J. (2023). Metabolomics Revealed Alterations in Serum Lipid Metabolite Levels in Childhood Atopic Dermatitis. Pediatr. Allergy Immunol..

[63] Ma E.Z., Deng J., Parthasarathy V., Lee K.K., Pritchard T., Guo S., Zhang C., Kwatra M.M., Le A., Kwatra S.G. (2024). Integrated Plasma Metabolomic and Cytokine Analysis Reveals a Distinct Immunometabolic Signature in Atopic Dermatitis. Front. Immunol..

[64] Yang L., Li D., Sun S., Liu D., Wang Y., Liu X., Zhou B., Nie W., Li L., Wang Y. (2024). Dupilumab Therapy Improves Gut Microbiome Dysbiosis and Tryptophan Metabolism in Chinese Patients with Atopic Dermatitis. Int. Immunopharmacol..

[65] Yu J., Chen T., Zhou H., Li S., Wu B., Xiong Y. (2025). Metabolomic Biomarkers and Altered Phenylalanine Metabolic Pathway in Preschool Children with Atopic Dermatitis, A Pilot Study. Indian J. Dermatol. Venereol. Leprol..

[66] Herr N., Bode C., Duerschmied D. (2017). The Effects of Serotonin in Immune Cells. Front. Cardiovasc. Med..

[67] Soga F., Katoh N., Inoue T., Kishimoto S. (2007). Serotonin Activates Human Monocytes and Prevents Apoptosis. J. Investig. Dermatol..

[68] Rasul A., El-Nour H., Lonne-Rahm S.B., Fransson O., Johansson C., Johansson B., Zubeidi M., Seeberg E., Djurfeldt D.R., Azmitia E. (2016). Serotonergic Markers in Atopic Dermatitis. Acta Derm. Venereol..

[69] de Mel S., Nordlind K., Holst M., Frohm-Nilsson M., Lonne-Rahm S.B. (2012). Polymorphisms in the Serotonin Transporter Gene of Patients with Atopic Dermatitis. Immunopharmacol. Immunotoxicol..

[70] Rausl A., Nordlind K., Wahlgren C.F. (2013). Pruritic and Vascular Responses Induced by Serotonin in Patients with Atopic Dermatitis. Acta Derm. Venereol..

[71] Kawana S., Kato Y., Omi T. (2010). Efficacy of a 5-HT1a Receptor Agonist in Atopic Dermatitis. Clin. Exp. Dermatol..

[72] Kiecka A., Szczepanik M. (2022). The Potential Action of SSRIs in the Treatment of Skin Diseases Including Atopic Dermatitis and Slow-Healing Wounds. Pharmacol. Rep..

[73] Jiang X., Wu X., Yang F., Li Y. (2025). Topical Application of Fluoxetine Improves DNCB-Induced Atopic Dermatitis in Mice. Exp. Dermatol..

[74] Jaworek A.K., Jaworek M., Makara-Studzińska M., Szafraniec K., Doniec Z., Szepietowski J., Wojas-Pelc A., Pokorski M. (2020). Depression and Serum Content of Serotonin in Adult Patients with Atopic Dermatitis. Adv. Exp. Med. Biol..

[75] Chang Y.S., Lin M.H., Lee J.H., Lee P.L., Dai Y.S., Chu K.H., Sun C., Lin Y.-T., Wang L.-C., Yu H.-H. (2016). Melatonin Supplementation for Children with Atopic Dermatitis and Sleep Disturbance: A Randomized Clinical Trial. JAMA Pediatr..

[76] Bešlić I., Lugović-Mihić L., Vrtarić A., Bešlić A., Škrinjar I., Hanžek M., Crnković D., Artuković M. (2023). Melatonin in Dermatologic Allergic Diseases and Other Skin Conditions. Int. J. Mol. Sci..

[77] Yang L., Wang P., Gao T., Huang X., Lin Z., Sweren E., Li Y., Chen L., Alphonse M.P., Zhang J. (2025). Melatonin Treatment Increases Skin Microbiota-Derived Propionic Acid to Alleviate Atopic Dermatitis. J. Allergy Clin. Immunol..

[78] Lavado-Fernández E., Pérez-Montes C., Robles-García M., Santos-Ledo A., García-Macia M. (2026). Melatonin at the Crossroads of Oxidative Stress, Immunity, and Cancer Therapy. Antioxidants.

[79] Wang P., Yang L., Chen L., Huang X., Lin Z., Li Y., Qi C., Xu T., Zhang J., Qi M. (2025). Phytic Acid Improves Skin Barrier Function in Murine Atopic Dermatitis via Skin Microbiota-Derived Indole-3-Propionic Acid. Allergy.

[80] Uberoi A., Murga-Garrido S.M., Bhanap P., Campbell A.E., Knight S.A.B., Wei M., Chan A., Senay T., Tegegne S., White E.K. (2025). Commensal-Derived Tryptophan Metabolites Fortify the Skin Barrier: A 50-Species Gnotobiotic Model of the Human Skin Microbiome. Cell Chem. Biol..

[81] Kim K., Kim H., Sung G.Y. (2022). Effects of Indole-3-Lactic Acid on IL-4/IL-13-Induced Human Skin-Equivalent Atopic Dermatitis Models. Int. J. Mol. Sci..

[82] Kang Y.M., Kim H.M., Lee J., Baek J.S., Lee M., An H.J. (2024). Indole-3-Carbinol Alleviates Allergic Skin Inflammation via Periostin/TSLP Suppression in Atopic Dermatitis. Chin. Med..

[83] Zhou H., Pei Z., Wang N., Fang Z., Hu W., Wang H., Lu W., Zhang H., Chen W. (2026). Specific Microbiota Tryptophan Metabolites, Indole-3-Aldehyde and Indole-3-Lactic Acid Are Key Mediators in Alleviating Atopic Dermatitis. Food Biosci..

[84] Rios-Carlos M., Cervantes-García D., Córdova-Dávalos L.E., Bermúdez-Humarán L.G., Salinas E. (2024). Unraveling the Gut–Skin Axis in Atopic Dermatitis. Gut Microbes.

[85] Wang F., Wang Z., Qu L. (2024). The Changes of Intestinal Flora and Metabolites in Atopic Dermatitis Mice. Front. Microbiol..

[86] Fang Z., Pan T., Wang H., Zhu J., Zhang H., Zhao J., Chen W., Lu W. (2022). Limosilactobacillus reuteri Attenuates Atopic Dermatitis via Changes in Gut Bacteria and Indole Derivatives. Int. J. Mol. Sci..

[87] Li Y., Li M., Ren Q., Ai C., Li S., Li H., Zhao S., Sui D., Ren X. (2025). Therapeutic Mechanism of Kynurenine, a Metabolite of Probiotics, on Atopic Dermatitis in Mice. Foods.

[88] Tan-Lim C.S.C., Esteban-Ipac N.A.R., Mantaring J.B.V., Yen E.C.S., Recto M.S.T., Sison O.T., Alejandria M.M. (2021). Comparative Effectiveness of Probiotic Strains for the Treatment of Pediatric Atopic Dermatitis: A Systematic Review and Network Meta-Analysis. Pediatr. Allergy Immunol..

[89] Zhang X., Huang X., Zheng P., Liu E., Bai S., Chen S., Pang Y., Xiao X., Yang H., Guo J. (2024). Changes in Oral, Skin, and Gut Microbiota in Children with Atopic Dermatitis: A Case-Control Study. Front. Microbiol..

[90] Wang M., Guo J., Hart A.L., Li J.V. (2023). Indole-3-Aldehyde Reduces Inflammatory Responses and Restores Intestinal Epithelial Barrier Function via AhR in Colitis Models. J. Inflamm. Res..

[91] Wang A., Guan C., Wang T., Mu G., Tuo Y. (2024). *Lactiplantibacillus plantarum*-Derived Indole-3-Lactic Acid Ameliorates Intestinal Barrier Integrity through AhR/Nrf2/NF-κB. J. Agric. Food Chem..

[92] Schjødt M.S., Gürdeniz G., Chawes B. (2020). The Metabolomics of Childhood Atopic Diseases: A Comprehensive Pathway-Specific Review. Metabolites.

[93] Peng J., Li Z., Wu W., Sun N., Yang X., Liu Q., Li H. (2026). Non-Invasive Detection of Pediatric Atopic Dermatitis Based on Fecal Microbiota and Metabolite Profiles: A Diagnostic Approach. Front. Immunol..

[94] Liu J., Xi K., Zhang L., Han M., Wang Q., Liu X. (2024). Tryptophan Metabolites and Gut Microbiota Play an Important Role in Pediatric Migraine Diagnosis. J. Headache Pain.

[95] Wazir A., O’Toole E.A. (2025). Itching for Innovation: The Role of Aryl Hydrocarbon Receptor Agonists as a Future Therapy for Atopic Dermatitis. Clin. Exp. Dermatol..

[96] Bissonnette R., Stein Gold L., Kircik L., Simpson E.L., Eichenfield L.F., Browning J., Hebert A.A., Alexis A.F., Soong W., Piscitelli S.C. (2025). Skin Clearance, Duration of Treatment-Free Interval, and Safety of Tapinarof Cream 1%: ADORING 3, 48-Week Phase 3 Open-Label Extension, Adults and Children ≥2 Years with Atopic Dermatitis. J. Am. Acad. Dermatol..

[97] Tsuji G., Yumine A., Yamamura K., Takemura M., Kido-Nakahara M., Ito T., Nakahara T. (2024). Tapinarof Regulates SEMA3A Expression in Human Keratinocytes through NRF2. J. Investig. Dermatol..

[98] Wang X., Mao D., Jia J., Zhang J. (2024). Benvitimod Inhibits IL-4/IL-13-Induced Tight Junction Impairment via AHR/ARNT and STAT6 Inhibition in Keratinocytes. J. Investig. Dermatol..

[99] Ahn Y.M., Shin S., Jang J.H., Jung J. (2023). Bojungikgi-Tang Improves Skin Barrier Function and Immune Response in Atopic Dermatitis Mice Fed a Low-AhR-Ligand Diet. Chin. Med..

[100] Proper S.P., Dwyer A.T., Appiagyei A., Felton J.M., Ben-Baruch Morgenstern N., Marlman J.M., Kotliar M., Barski A., Troutman T.D., Rothenberg M.E. (2024). AhR and IL-13 Signaling Crosstalk in Human Keratinocytes and Atopic Dermatitis. Front. Allergy.

[101] Cha J., Kim T.-G., Bhae E., Gwak H.-J., Ju Y., Choe Y.H., Jang I.-H., Jung Y., Moon S., Kim T. (2024). Skin Microbe-Dependent TSLP-ILC2 Priming Axis in Early Life Is Co-Opted in Allergic Inflammation. Cell Host Microbe.

[102] Jiang Z., Jhun J., Ryu J.-G., Choi J.-Y., Kang S.-J., Kwon J.-Y., Choi J.-W., Ryu H., Jung K., Choi S. (2022). Accurate Diagnosis of Atopic Dermatitis by Combining Transcriptome and Microbiota Data with Supervised Machine Learning. Sci. Rep..

[103] Zhakparov D., Lunjani N., Schmid M., Moriarty K., Roquero D., Dreher A., Heldstab-Kast J.I., Nadeau K.C., Akdis C., Levin M. (2026). Protective and Susceptibility Clusters of Environmental Factors, Gene Expression, Antibody Responses, and Cytokines in Pediatric Atopic Dermatitis: Insights from Multi-Modal Data Integration. medRxiv.

[104] Bocheva G.S., Slominski R.M., Slominski A.T. (2021). Immunological Aspects of Skin Aging in Atopic Dermatitis. Int. J. Mol. Sci..

[105] Van der Leek A.P., Yanishevsky Y., Kozyrskyj A.L. (2017). The Kynurenine Pathway as a Novel Link between Allergy and the Gut Microbiome. Front. Immunol..

