# Supplementary material for: Tryptophan Metabolism in Pediatric Atopic Dermatitis: Shared Mechanisms, Age-Specific Biomarkers, and Emerging Therapies
Source: Biomedicines. 2026 Sep 22;14(10):2140. doi: 10.3390/biomedicines14102140 (PMC13701361; doi:10.3390/biomedicines14102140)
Supplement: Supplementary file 1 [file biomedicines-14-02140-s001.zip › biomedicines-4567156-supplementary.pdf]

Supplementary Table S1. Classification of reviewed literature

Full classification of all references cited in this review, generated during the literature search described in Section 1.4 (Literature Search Methodology). Age band reflects the population or model studied (human age in years unless otherwise noted). Role categories: BACKGROUND = contextual/background reference; CORE-P = core pediatric evidence (<18 years); CORE-A = core adult evidence (≥18 years); CORE-M = core evidence spanning/comparing multiple ages; MECH = mechanistic/preclinical (animal or in vitro) evidence; THER = therapeutic evidence; INDIRECT = indirectly related evidence. An asterisk (*) marks references also identified as key primary evidence in the main text.

ID	Population / model	Age	AD-related	Main domain	Evidence type	Role
**1**	Pediatric AD	<18	Yes	Molecular biology	Review	BACKGROUND
**2**	Pediatric AD	<18	Yes	Epidemiology	Human	CORE-P
**3**	AD	Mixed	Yes	Epidemiology	Review	BACKGROUND
**4**	AD	Mixed	Yes	Prevalence	SR/meta-analysis	BACKGROUND
**5**	Pediatric AD	<18	Yes	Prevalence	SR/meta-analysis	BACKGROUND
**6**	Pediatric AD	<18	Yes	Disease burden	Human	CORE-P
**7**	Pediatric AD	<18	Yes	Epidemiology	Human	CORE-P
**8**	Pediatric + adult AD	Mixed	Yes	Global burden	Review	BACKGROUND
**9**	AD	Mixed	Yes	TH2/TH17 endotype	Human	CORE-M
**10**	AD	Mixed	Yes	Endotypes	Review	BACKGROUND
**11**	AD	Mixed	Yes	Biomarkers	Review	BACKGROUND
**12**	AD	Mixed	Yes	Serum biomarkers/endotypes	Human	CORE-M
**13**	AD	Mixed	Yes	Precision medicine	Review	BACKGROUND
**14**	AD	Mixed	Yes	Precision medicine	Review	BACKGROUND
**15**	Pediatric AD	<18	Yes	TH2/TH17	Human	CORE-P
**16**	Pediatric AD	<18	Yes	TH2/TH17/TH22	Human	CORE-P
**17**	AD	Multiple ages	Yes	Molecular age signatures	Human	CORE-M
**18**	AD	Multiple ages	Yes	T-cell subsets/age	Human	CORE-M
**19**	AD	Mixed	Yes	Biomarkers	Review	BACKGROUND
**20**	AD	Mixed	Yes	Tryptophan metabolism	Review	BACKGROUND
**21**	AD	Mixed	Yes	Filaggrin/skin barrier	Review	BACKGROUND
**22**	Early-life AD	<18	Yes	FLG/skin barrier	Human	CORE-P
**23**	Infants	<18	Yes	FLG/skin barrier	Human	CORE-P
**24**	AD	Multiple ages	Yes	Immunophenotype	Human	CORE-M
**25**	Infants	<18	Yes	Gut microbiome	Human	CORE-P

ID	Population / model	Age	AD-related	Main domain	Evidence type	Role
**26**	Infants/eczema	<18	Yes	Fecal tryptophan metabolism	Prospective human	CORE-P *
**27**	Pregnancy/offspring	Maternal + infant	Related	Tryptophan/immune programming	Human	INDIRECT
**28**	Pregnancy/offspring	Maternal + infant	Yes	Nicotinamide metabolites	Human	INDIRECT
**29**	AD	Mixed	Yes	Immunology/aging	Review	BACKGROUND
**30**	Children	<18	Yes	TARC/CCL17	Human	CORE-P
**31**	Infants	<18	Yes	Severity biomarkers	Human	CORE-P
**32**	Infants	<18	Yes	TARC/severity	Human	CORE-P
**33**	Children	<18	Yes	Chemokines	Human	CORE-P
**34**	Children	<18	Yes	miRNA-155	Human	CORE-P
**35**	Children	<18	Yes	miRNA	Human	CORE-P
**36**	Pediatric alopecia +/- AD	<18	Partly	Immune biomarkers	Human	INDIRECT
**37**	General human biology	Mixed	No	Tryptophan	Review	BACKGROUND
**38**	General human biology	Mixed	No	Tryptophan/serotonin/kynurenine	Review	BACKGROUND
**39**	General biology	Mixed	No	Tryptophan metabolism	Review	BACKGROUND
**40**	Host + microbiome	Mixed	Related	Tryptophan metabolism	Review	BACKGROUND
**41**	Inflammation/infection	Mixed	Indirect	Kynurenine	Review	INDIRECT
**42**	Immune system	Mixed	Indirect	IDO1	Review	BACKGROUND
**43**	Immune system	Mixed	Indirect	Tryptophan/immune regulation	Review	BACKGROUND
**44**	AD model	Experimental	Yes	Microbial indoles/AHR	Experimental	MECH *
**45**	Microbiota/immune system	Experimental	Related	Indoles/AHR/IL-22	Experimental	MECH
**46**	AD mouse	Animal	Yes	B. longum/tryptophan	Experimental	MECH *
**47**	Intestinal microbiota	Mixed	Related	Indoles	Review	BACKGROUND
**48**	Immune system	Mixed	Related	AHR ligands	Review	BACKGROUND
**49**	Skin/AD	Mixed	Yes	AHR	Review	BACKGROUND
**50**	AD/psoriasis	Mixed	Yes	AHR	Review	BACKGROUND
**51**	Skin microbiome	Experimental	Yes	AHR/skin barrier	Experimental	MECH *
**52**	Mouse skin	Animal	Related	Keratinocyte AHR	Experimental	MECH
**53**	Immune modulation	Mixed	Related	AHR therapy	Review	BACKGROUND
**54**	AD	Mixed	Yes	AHR/natural products	Review	BACKGROUND
**55**	General biology	Mixed	Indirect	Kynurenine metabolites	Review	BACKGROUND
**56**	General biology	Mixed	Indirect	Kynurenine pathway	Review	BACKGROUND
**57**	General biology	Mixed	Indirect	Tryptophan homeostasis	Review	BACKGROUND
**58**	Inflammatory disease	Mixed	Indirect	Kynurenine/AHR	Review	BACKGROUND

ID	Population / model	Age	AD-related	Main domain	Evidence type	Role
**59**	Dendritic cells	Experimental	Indirect	L-kynurenine	Experimental	MECH
**60**	Psoriasis/inflammatory disease	Mixed	Indirect	Kynurenine pathway	Experimental	INDIRECT
**61**	Children/allergic diseases	<18	Related	IDO activity/biomarkers	Human	CORE-P *
**62**	Immune cells	Mixed	Indirect	Serotonin	Review	BACKGROUND
**63**	Human monocytes	Mixed	Indirect	Serotonin/immune response	Experimental	MECH
**64**	AD	>=18	Yes	Serotonergic markers	Human	CORE-A
**65**	AD	>=18	Yes	Serotonin transporter	Human	CORE-A
**66**	AD	>=18	Yes	Serotonin/pruritus	Human	CORE-A
**67**	AD	>=18	Yes	5-HT1A therapy	Clinical	THER/CORE-A
**68**	AD/skin diseases	Mixed	Yes	SSRI/therapy	Review	BACKGROUND
**69**	AD mouse	Animal	Yes	Fluoxetine	Experimental	THER/MECH
**70**	AD	>=18	Yes	Serum serotonin	Human	CORE-A
**71**	Children with AD	<18	Yes	Melatonin/sleep	RCT	THER/CORE-P
**72**	Allergic skin disease	Mixed	Related	Melatonin	Review	BACKGROUND
**73**	AD model	Experimental	Yes	Melatonin/microbial metabolites	Experimental	THER/MECH *
**74**	General biology	Mixed	Indirect	Melatonin/immunity	Review	BACKGROUND
**75**	AD mouse	Animal	Yes	IPA/skin barrier	Experimental	MECH *
**76**	Skin microbiome model	Experimental	Related/AD-relevant	Microbial tryptophan metabolites	Experimental	MECH *
**77**	AD skin-equivalent model	In vitro	Yes	Indole-3-lactic acid	Experimental	MECH *
**78**	AD model	Experimental	Yes	Indole-3-carbinol/AHR-related	Experimental	MECH *
**79**	AD model	Experimental	Yes	I3A/ILA	Experimental	MECH *
**80**	AD/gut-skin axis	Mixed	Yes	Microbiome	Review	BACKGROUND
**81**	AD mouse	Animal	Yes	Gut microbiota/metabolites	Experimental	MECH
**82**	AD mouse	Animal	Yes	L. reuteri/indole derivatives	Experimental	MECH *
**83**	AD mouse	Animal	Yes	Kynurenine	Experimental	MECH *
**84**	Pediatric AD	<18	Yes	Probiotics	SR/NMA	BACKGROUND
**85**	Children with AD	<18	Yes	Gut/skin/oral microbiome	Human	CORE-P *
**86**	Colitis model	Experimental	Indirect	I3A/AHR/barrier	Experimental	INDIRECT
**87**	Intestinal model	Experimental	Indirect	ILA/AHR/barrier	Experimental	INDIRECT
**88**	Childhood atopic disease	<18	Related	Metabolomics	Review	BACKGROUND
**89**	Pediatric AD	<18	Yes	Fecal microbiome/metabolites	Human	CORE-P *
**90**	Childhood AD	<18	Yes	Serum metabolomics/eicosanoids	Human	CORE-P *
**91**	Childhood AD	<18	Yes	Lipid metabolomics	Human	CORE-P *
**92**	AD	Mixed	Yes	Plasma metabolomics/cytokines	Human	CORE-M

ID	Population / model	Age	AD-related	Main domain	Evidence type	Role
**93**	AD	>=18	Yes	Dupilumab/microbiome/tryptophan	Human	CORE-A/THER *
**94**	Preschool AD	<18	Yes	Metabolomic biomarkers	Human	CORE-P *
**95**	Children with migraine	<18	No/indirect	Tryptophan/microbiome	Human	INDIRECT
**96**	AD	Mixed	Yes	AHR agonists	Review	BACKGROUND
**97**	AD	>=2 years	Yes	Tapinarof/AHR	Clinical trial	THER *
**98**	Keratinocytes	In vitro	Yes	Tapinarof/NRF2	Experimental	MECH/THER *
**99**	Keratinocytes/AD	In vitro	Yes	AHR/ARNT/STAT6	Experimental	MECH *
**100**	AD mouse	Animal	Yes	AHR-ligand diet	Experimental	MECH *
**101**	AD keratinocytes	In vitro	Yes	AHR/IL-13	Experimental	MECH *
**102**	Early-life allergic inflammation	Experimental	Related	Skin microbiome/TSLP/ILC2	Experimental	MECH
**103**	AD	Mixed	Yes	Transcriptome/microbiome/ML	Human/diagnostic	CORE-M *
**104**	Pediatric AD	<18	Yes	Multimodal biomarkers	Preprint	CORE-P *
**105**	Allergy/gut microbiome	Mixed	Related	Kynurenine pathway	Review	BACKGROUND
